# Household Income and Offspring Education Explain Blacks’ Diminished Returns of Parental Education

**DOI:** 10.31586/ojp.2024.986

**Published:** 2024-07-18

**Authors:** Shervin Assari, Hossein Zare

**Affiliations:** 1 Department of Internal Medicine, Charles R. Drew University of Medicine and Science, Los Angeles, CA, United States; 2 Department of Family Medicine, Charles R. Drew University of Medicine and Science, Los Angeles, CA, United States; 3 Department of Urban Public Health, Charles R. Drew University of Medicine and Science, Los Angeles, CA, United States; 4 Marginalization-Related Diminished Returns (MDRs) Center, Los Angeles, CA, United States; 5 Department of Health Policy and Management, Johns Hopkins Bloomberg School of Public Health, Baltimore, MD, United States; 6 School of Business, University of Maryland Global Campus (UMGC), College Park, MD, United States

**Keywords:** Parental Education, Minorities’ Diminished Returns, Racial Disparities, Household Income, Educational Attainment

## Abstract

**Background:**

High parental education promotes various aspects of offspring well-being including reducing their risk of depression/anxiety, criminal justice involvement, and welfare reliance. However, according to minorities’ diminished returns, these benefits are not equal across racial groups, with Black families experiencing diminished returns of parental education compared to White families. This study explores the role of household income and offspring educational attainment as potential serial pathways that operate as mechanisms underlying diminished returns of parental education on offspring outcomes in Black families. Gender differences in these effects were also explored.

**Methods:**

Utilizing data from the Future of Families and Child Wellbeing Study (FFCWS) over a 22-year follow-up period (seven waves), we examined the serial mediation by household income and offspring educational attainment in explaining the relationship between parental education and offspring outcomes namely depression, anxiety, criminal justice involvement, and welfare reliance [Temporary Assistance for Needy Families (TANF) and Supplemental Nutrition Assistance Program (SNAP)]. We used structural equation modeling (SEM) with household income as the first mediator and young adult education as the second mediator. Multi-group models were used to explore gender differences in these paths.

**Results:**

The study confirmed the role of our proposed serial mediators for Blacks’ weaker effects of parental education on offspring outcomes. We observed weaker effects of first affects household income, with this effect being for Black families compared to White families, which then impacted educational attainment of the offspring. The findings indicate that household income plays a crucial mediating role, but its effect is weaker in Black families. Additionally, the educational attainment of offspring from highly educated Black parents is less effective in improving outcomes compared to their White peers, further contributing to diminished returns. Some gender differences were observed for the effects of educational attainment on economic and health outcomes of young adults.

**Conclusions:**

The study underscores the need to reconsider traditional assumptions about the comparability of family conditions and outcomes across racial groups with similar levels of parental education. The findings highlight the importance of targeted policies and interventions aimed at enhancing the economic stability and educational outcomes of Black families to address these disparities. Policies should focus on promoting the economic well-being of highly educated Black parents and improving the educational outcomes of their children.

## 1. Introduction

Parental education significantly impacts various aspects of offspring well-being, including physical and mental health, criminal justice involvement, and reliance on welfare [1, 2]. Generally, children of highly educated parents are less likely to suffer from anxiety and depression, are less likely to be incarcerated, and are less dependent on social welfare programs [3–6]. However, these beneficial effects of parental education are not equal across racial groups [7–9]. According to the Minorities’ Diminished Returns (MDRs) theory[10], the positive effects of resources like parental education are often less pronounced in marginalized families, such as Black families, compared to their White counterparts[11–14]. This suggests that the benefits of parental education on offspring outcomes, such as reducing anxiety [15], depression [16], or poverty [7], are weaker among Black families.

Several studies have documented these diminished returns [12, 13, 17–23], however, the pathways through which parental education impacts offspring outcomes are likely complex and multifaceted [11, 12, 24, 25]. One potential mechanism is the lower household income in Black families, as the translation of parental education into economic benefits is often less effective in these families compared to White families [26–29]. This is because highly educated Black people work in worse occupations than highly educated white people [30–33]. Another possible mechanism is the educational attainment of the offspring, where offspring from highly educated Black parents may still experience worse outcomes compared to their White peers [17].

The aim of this study is to investigate how household income and offspring educational attainment serve as pathways through which the reduced benefits of parental education on offspring outcomes emerge among Black families. Utilizing data from the Future of Families and Child Wellbeing Study (FFCWS) [34–38] over a 22-year follow-up period, this study investigates how these factors contribute to the differential impacts of parental education at birth on offspring outcomes when they are 22-year-old between Black and White racial groups.

## 2. Methods

### 2.1. Design and Setting

The Future of Families and Child Wellbeing Study (FFCWS), formerly known as the Fragile Families and Child Wellbeing Study, is a pioneering research project aimed at understanding the challenges faced by economically disadvantaged families in the United States [35, 39–42]. The FFCWS follows a birth cohort starting in 1998-2000, tracking children from birth to young adulthood at age 22 in 2000-2022. Detailed information on the study’s sampling techniques and methodologies can be found in previously published literature. This section provides a brief overview of the FFCWS research design.

### 2.2. Ethics

The study protocol was approved by the Institutional Review Board at Princeton University. Informed consent was obtained from all participating families, with parents or legal guardians consenting on behalf of minors, who also provided their assent. All data collection, storage, and analysis procedures were designed to protect participants’ anonymity, and families were compensated for their participation.

### 2.3. Sample and Sampling

The FFCWS recruited a diverse sample of urban families from 20 major U.S. cities, each with a population exceeding 200,000. The study specifically targeted underrepresented families, particularly non-married, Black, and Hispanic families. Consequently, the study’s sample predominantly consists of low socioeconomic status families, with a substantial representation of Black and Hispanic participants, which does not reflect the overall U.S. population. The analytical sample included 1,264 families with a Black father and son, excluding families where the child was female, or the parents were identified as White or Latino.

### 2.4. Process

Our analysis utilized data from the first and seventh waves of the FFCWS. Socioeconomic status (SES) data were collected at birth (wave 1, year 1998/2000, baseline), and outcomes were measured when the offspring were young adults 22 years later (wave 7, year 2020/2022, follow up). The analysis included 2,500 Black and White families with follow-up data.

### 2.5. Predictors

Data were collected through interviews with both parents, covering parents’ education, age, family structure at birth, and fathers’ incarceration history. Collecting data from both parents was essential to ensure accurate reporting of sensitive information, such as criminal history, which might be underreported if only gathered from the male partner. Education levels of both mother and father were assessed at the study’s outset and categorized into four levels: (1) less than high school, (2) high school diploma, (3) some college, and (4) college degree or higher.

### 2.6. Outcomes

At age 22, the FFCWS measured various outcomes for the children, including educational attainment, incarceration history, welfare use [Temporary Assistance for Needy Families (TANF) and Supplemental Nutrition Assistance Program (SNAP)], and mental health. Mental health status was determined based on self-reported medical diagnoses of anxiety and depression (two items). Educational attainment, incarceration history, and welfare use were self-reported by the participants.

### 2.7. Statistical Analysis

Data analysis was conducted using STATA version 18.0. For the multivariable analysis, we applied structural equation modeling (SEM) to examine the effects of family demographic and SES factors as predictors on youth outcomes 22 years later. Structural Equation Modeling (SEM) is a powerful statistical technique that offers distinct advantages for testing mediation and conducting longitudinal analyses in research. SEM allows researchers to examine complex relationships among variables by simultaneously estimating multiple regression equations within a single model. This capability is particularly beneficial for mediation analysis, where SEM can assess the direct and indirect effects of variables on outcomes through intermediate variables. In longitudinal studies, SEM enables the exploration of how variables change over time and how these changes are interrelated. By incorporating latent variables and measurement error into models, SEM provides more accurate estimates and enhances the understanding of underlying theoretical frameworks. Its ability to handle missing data effectively also makes SEM well-suited for longitudinal designs where attrition or incomplete data are common. Overall, SEM facilitates a comprehensive approach to investigating mediation processes and longitudinal dynamics, offering researchers a robust tool to uncover intricate relationships and mechanisms over extended time periods. The analysis explored the impact of household income at birth as the first mediator and offspring educational attainment (at age 22) as the second mediator. We had offspring anxiety, depression, SNAP, TANF, and incarceration history (all measured at age 22) as outcomes.

## 3. Results

### 3.1. Overall results

**Figure 1.**
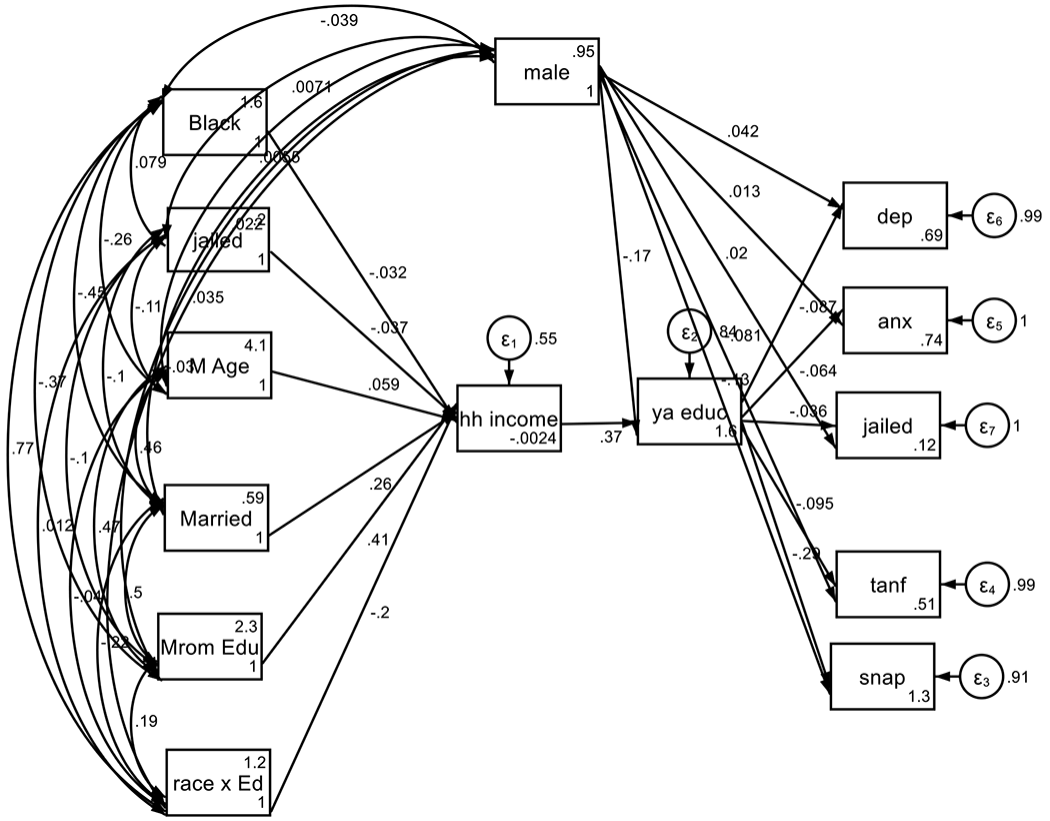
Summary of SEM with two mediators

The study confirmed the role of two serial mediators as serial mechanisms for Blacks’ diminished returns of parental education on youth outcomes 22 years later. We found that parental education first affects household income, however, this effect is weaker for Black families compared to White families. We also found that household income subsequently influences the educational attainment of the offspring. Thus, while household income plays a crucial mediating role in carrying the effects of parental education on youth outcomes, this effect is weaker in Black than White families. Therefore, the educational attainment of offspring carries the effect of highly educated parents on improving outcomes. These mechanisms contribute to diminished returns of parental education on youth outcomes in Black families (Figure 1).

### 3.2. Gender-Stratified Results

**Figure 2.**
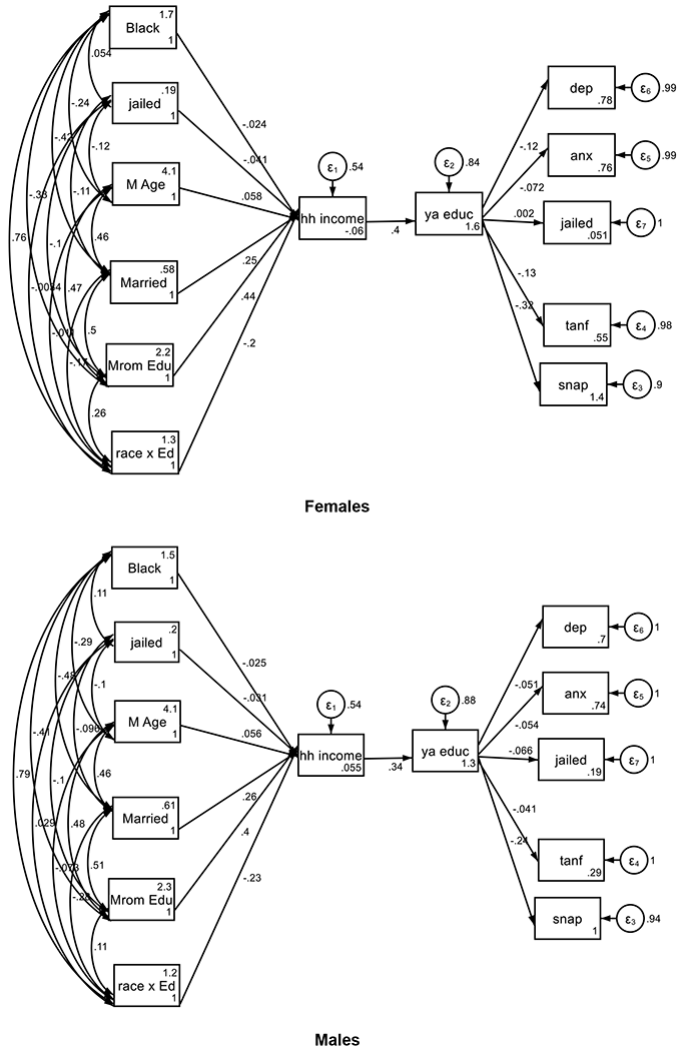
Summary of SEM with two mediators in females and males

A few gender differences were observed for the effects of educational attainment on young adult outcomes. The protective effects of young adult educational attainment on depression and anxiety were significant for females not males. In contrast, the protective effect of young adult educational attainment on being jailed was significant for males but not females (Figure 2, Table 2).

## 4. Discussion

The primary aim of this study was to investigate the mechanisms underlying the diminished returns of parental education on offspring outcomes among Black families compared to White families. Specifically, we focused on household income and offspring educational attainment as potential serial pathways that mediating differential returns of parental education on young adults’ health and economic outcomes. Using the FFCWS data over a 22-year follow-up period, we found that weaker effects of parental education on offspring outcomes in Black families is mediated through two serial mechanisms: lower household income at birth followed by lower educational attainment of the young offspring. Some gender differences were observed for the effects of educational attainment on economic and health outcomes of young adults.

Household income plays a crucial role in mediating the effects of parental education on offspring outcomes. Higher parental education is typically associated with increased household income, which in turn leads to better offspring outcomes. This effect is in part because occupation improves as education enhances. However, the effect of education on income is weaker for Black families, where the same level of parental education does not translate into comparable high paying jobs that result in comparable economic benefits as it does for White families. This racial disparity in the effect of parental education on household income is the first step that society reduces the positive impact of parental education on offspring outcomes in Black compared to White families.

Lower offspring educational attainment is the second mechanism through which the effects of parental education and household income become smaller on offspring health and economic outcomes in Black families. Higher parental education generally leads to better educational outcomes for offspring, which further improves their overall well-being. However, Black offspring with highly educated parents still face educational, societal, and other barriers that limit their educational aspirations and opportunities, leading to spillover effects on various subsequent life outcomes compared to their White peers. This diminished educational attainment among Black offspring from highly educated families is the second mechanism that weakens the positive effects of parental education on their outcomes in Black families.

These two mechanisms are not the only mechanisms that explain diminished returns of parental education in Black families. Other mechanisms related to several structural, historical, and societal factors may explain these diminished returns. Black parents, even with similar educational levels as White parents, are more likely to work in lower-quality jobs, experience higher levels of stress, and live in less favorable neighborhoods. These adverse conditions limit the ability of parental education to produce the same beneficial effects in Black families as in White families.

We identified gender differences in the mechanisms that explain how minorities experience reduced benefits from parental education on youth outcomes through income and young adult educational attainment. The protective effects of young adult educational attainment on depression and anxiety were significant for females not males. In contrast, the protective effect of young adult educational attainment on being jailed was significant for males but not females. Male and female Black young adults face distinct risks in the outcomes studied here. Racism and sexism intersect in complex ways, shaping unique experiences for Black boys, girls, young men, and women. Black females often find themselves more frequently heading single-parent households and starting childbearing at younger ages, which entails distinctive challenges and responsibilities. In contrast, Black men, while often fathers, are more likely to be non-resident and may not consistently share the same parenting responsibilities as Black mothers. These differing experiences are influenced by various interconnected mechanisms, including societal stereotypes, economic disparities, and historical injustices. The racialization of sexism means that Black women encounter gender discrimination influenced by their racial identity, while gendered racism inflicts distinct forms of discrimination on Black men. These dynamics underscore the necessity for nuanced approaches to address the intersecting oppressions faced by Black communities, aiming to foster equity and dismantle systemic barriers that perpetuate these disparities.

### 4.1. Research Implications

Future research should explore additional factors that contribute to the diminished returns of parental education in marginalized communities. This includes examining the role of state policies, social support systems, and other structural determinants that could help mitigate these disparities and promote equitable outcomes. Cultural barriers such as stigma and financial literacy may also have a role. Lower educational quality, labor market discrimination, segregation, and lower educational aspirations may also have some roles. Most importantly, some of these pathways may be gender specific.

### 4.2. Implications

The findings of this study highlight the need to reconsider the assumption that families with similar levels of parental education experience comparable conditions and outcomes across racial groups. Policymakers and researchers must recognize the unique challenges faced by Black families, even among those with high levels of parental education. To address transgenerational disparities among middle class families, policies should aim to enhance the economic stability of highly educated Black families and improve the educational outcomes of their children. These policies require enhancing quality of education and dismantling labor market discrimination in majority Black communities. There is also a need to reduce segregation so that middle-class Black families can leverage their human capital to ascend the social ladder and accumulate wealth. Interventions could include targeted economic support, job quality improvements, and educational resources tailored to the specific needs of these families.

### 4.3. Limitations and strengths

This study has several limitations. The FFCWS data, while comprehensive, does not capture all relevant variables influencing the outcomes studied here. Any long-term follow-up study such as the FFCWS is prone to bias due to differential attrition. Additionally, the study focuses on specific mechanisms and may not account for other potential pathways through which parental education affects offspring outcomes. Our analysis only included Black and White families, and all variables were collected at the individual or family level, lacking data on neighborhood, community, or policy levels. Finally, the sample size was not balanced between Black and White families. Long-term studies like the FFCWS encounter numerous challenges due to their extended follow-up durations. These challenges include evolving family dynamics and SES over time, alongside concerns about attrition and its potentially uneven impact across SES and racial demographics. These factors can significantly impact the study’s integrity and the interpretation of its findings. Changes in family dynamics, such as shifts in household composition or economic circumstances, were not systematically measured, introducing variability that complicates longitudinal analyses. Similarly, fluctuations in SES can alter participants’ access to resources and opportunities, potentially influencing outcomes measured over the study period. Attrition is another critical concern, with differing rates among SES and racial groups potentially skewing samples and compromising the study’s ability to generalize findings. Addressing these challenges necessitates robust methodologies to track participant changes, deliberate strategies to mitigate attrition biases, and a nuanced understanding of how socioeconomic and racial factors interact with study variables across the longitudinal timeline. Despite these limitations, the study’s strengths include its long follow-up period and the diversity of its sample of fragile families.

## 5. Conclusions

Our study findings underscore the complex mechanisms that underlie the diminished returns of parental education on offspring outcomes in Black families. By identifying lower household income and subsequent lower educational attainment of offspring as key pathways, we highlight the need for targeted interventions, policies, and programs to address these disparities and promote more equitable outcomes for all families. Implementing policies to increase the minimum wage and enhancing educational opportunities for Black youth are among the major strategies that may help equalize the returns of parental education between Black and White families.

## Figures and Tables

**Table 1.** Summary of SEM with two mediators

		Beta	SE	95% CI		p
HH Income / 10000 at Birth	Paternal Incarceration at Birth	−0.04	0.02	−0.07	0.00	0.031
	Maternal Education (1-4) at Birth	0.41	0.03	0.35	0.48	< 0.001
	Race (Black)	−0.03	0.05	−0.13	0.06	0.510
	Maternal Age at Birth	0.06	0.02	0.02	0.10	0.004
	Race (Black) x Parental Education at Birth	−0.20	0.05	−0.29	−0.11	< 0.001
	Married HH at Birth	0.26	0.02	0.21	0.30	< 0.001
	Intercept	0.00	0.11	−0.22	0.22	0.983
YA Education (1-4) at Age 22	HH Income / 10000 at Birth	0.37	0.02	0.33	0.41	< 0.001
	Gender (Male)	−0.17	0.02	−0.22	−0.13	< 0.001
	Intercept	1.58	0.05	1.49	1.68	< 0.001
YA SNAP at Age 22	YA Education (1-4) at Birth	−0.29	0.02	−0.33	−0.25	< 0.001
	Gender (Male)	−0.13	0.02	−0.18	−0.09	< 0.001
	Intercept	1.33	0.05	1.23	1.43	< 0.001
YA TANF at Age 22	YA Education (1-4)	−0.09	0.02	−0.14	−0.05	< 0.001
	Gender (Male)	−0.08	0.02	−0.13	−0.04	0.001
	Intercept	0.51	0.06	0.40	0.61	< 0.001
YA Anxiety at Age 22	YA Education (1-4)	−0.06	0.03	−0.11	−0.01	0.011
	Gender (Male)	0.01	0.03	−0.04	0.06	0.605
	Intercept	0.74	0.06	0.62	0.86	< 0.001
YA Depression at Age 22	YA Education (1-4)	−0.09	0.02	−0.14	−0.04	< 0.001
	Gender (Male)	0.04	0.03	−0.01	0.09	0.099
	Intercept	0.69	0.06	0.57	0.81	< 0.001
YA Jailed at Age 22	YA Education (1-4)	−0.04	0.02	−0.08	0.01	0.140
	Gender (Male)	0.02	0.02	−0.03	0.07	0.386
	Intercept	0.12	0.06	0.00	0.23	0.046

Temporary Assistance for Needy Families (TANF); Supplemental Nutrition Assistance Program (SNAP); Young Adult (YA); Household (HH)

**Table 2.** Summary of SEM with two mediators in females and males

		Beta	SE	95% CI		p
						
**Females**						
HH Income/ 10000						
	Paternal Incarceration at Birth	−0.04	0.02	−0.09	0.01	0.082
	Maternal Education (1-4) at Birth	0.44	0.05	0.35	0.54	< 0.001
	Race (Black)	−0.02	0.06	−0.15	0.10	0.707
	Maternal Age at Birth	0.06	0.03	0.00	0.11	0.038
	Race x Parental Education (1-4)	−0.20	0.06	−0.33	−0.07	0.002
	Married HH at Birth	0.25	0.03	0.20	0.31	< 0.001
	Intercept	−0.06	0.15	−0.36	0.24	0.699
						
YA Education at Age 22 (1-4)						
	HH Income/ 10000	0.40	0.03	0.34	0.45	< 0.001
	Intercept	1.57	0.06	1.44	1.69	< 0.001
						
YA SNAP at Age 22						
	YA Education (1-4)	−0.32	0.03	−0.38	−0.27	< 0.001
	Intercept	1.38	0.06	1.26	1.50	< 0.001
						
YA TANF at Age 22						
	YA Education (1-4)	−0.13	0.03	−0.19	−0.07	< 0.001
	Intercept	0.55	0.07	0.41	0.68	< 0.001
						
YA Anxiety at Age 22						
	YA Education (1-4)	−0.07	0.03	−0.14	0.00	0.036
	Intercept	0.76	0.08	0.61	0.91	< 0.001
						
YA Depression at Age 22						
	YA Education (1-4)	−0.12	0.03	−0.19	−0.05	< 0.001
	Intercept	0.78	0.08	0.63	0.93	< 0.001
						
YA Jailed at Age 22						
	YA Education (1-4)	0.00	0.03	−0.06	0.06	0.950
	Intercept	0.05	0.07	−0.09	0.19	0.466
						
**Males**						
HH Income/ 10000 at Birth						
	Paternal Incarceration at Birth	−0.03	0.03	−0.08	0.02	0.211
	Maternal Education (1-4) at Birth	0.40	0.05	0.30	0.49	< 0.001
	Race (Black)	−0.02	0.07	−0.17	0.12	0.730
	Maternal Age at Birth	0.06	0.03	0.00	0.11	0.055
	Race x Parental Education (1-4)	−0.23	0.07	−0.36	−0.10	0.001
	Married HH at Birth	0.26	0.03	0.19	0.32	< 0.001
	Intercept	0.05	0.16	−0.27	0.38	0.738
						
YA Education (1-4) at Age 22						
	HH Income/ 10000 at Birth	0.34	0.03	0.28	0.40	< 0.001
	Intercept	1.29	0.06	1.17	1.42	< 0.001
						
YA SNAP at Age 22						
	YA Education (1-4) at Age 22	−0.24	0.03	−0.30	−0.18	< 0.001
	Intercept	1.02	0.06	0.90	1.14	< 0.001
						
YA TANF at Age 22						
	YA Education (1-4) at Age 22	−0.04	0.03	−0.11	0.03	0.225
	Intercept	0.29	0.07	0.16	0.42	< 0.001
						
YA Anxiety at Age 22						
	YA Education (1-4) at Age 22	−0.05	0.04	−0.12	0.02	0.133
	Intercept	0.74	0.07	0.60	0.88	< 0.001
						
YA Depressed at Age 22						
	YA Education (1-4) at Age 22	−0.05	0.04	−0.12	0.02	0.151
	Intercept	0.70	0.07	0.56	0.84	< 0.001
						
YA Jailed at Age 22						
	YA Education (1-4) at Age 22	−0.07	0.04	−0.14	0.00	0.067
	Intercept	0.19	0.07	0.06	0.32	0.005

Temporary Assistance for Needy Families (TANF); Supplemental Nutrition Assistance Program (SNAP); Young Adult (YA); Household (HH)

## Data Availability

The FFCWS data are available to public at Office of Population Research data repository available at https://oprdata.princeton.edu/archive/restricted/Default.aspx.
